# Study on medication adherence in patients with gestational diabetes mellitus complicated by postpartum glucose metabolism disorders based on the risk perception attitude framework

**DOI:** 10.3389/fendo.2025.1501541

**Published:** 2025-05-15

**Authors:** Bing Zhou, Zhuolin Zhou, Yu Sun, Ting He, Weihong Ge, Lingjun Sun, Cheng Ji

**Affiliations:** ^1^ Department of Pharmacy, Nanjing Drum Tower Hospital Clinical College of Nanjing University of Chinese Medicine, Nanjing, China; ^2^ Department of Pharmacy, China Pharmaceutical University Nanjing Drum Tower Hospital, Nanjing, China; ^3^ Department of Pharmacy, Nanjing Drum Tower Hospital, Affiliated Hospital of Medical School, Nanjing University, Nanjing, China

**Keywords:** risk perception attitude framework theory, gestational diabetes mellitus, postpartum glucose metabolism abnormalities, medication adherence, blood glucose management

## Abstract

**Objective:**

This study aims to assess the compliance with anti-hypoglycemic drugs among postpartum patients with gestational diabetes mellitus (GDM) through a questionnaire survey, and to examine the factors that may influence this adherence. The goal is to enhance pharmacists’ medication practices and improve glycemic control.

**Method:**

Based on the theory of risk perception attitude framework, the study selected pregnant women who delivered at Nanjing DrumTower Hospital’s obstetrics department from 2020 to 2022 and were diagnosed with GDM as the research subjects. The Morisky Medication Adherence Scale was used to evaluate their medication adherence. The correlation matrix was used to express whether there is a correlation between risk perception, self-efficacy, and medication adherence. Finally, a linear hierarchical regression model was used to present the moderating effect of self-efficacy on the relationship between risk perception and medication adherence.

**Results:**

A total of 80 postpartum GDM patients receiving medication intervention were included in the study to assess medication adherence. The median score on the MMAS-8 scale was 3.75 (3.50, 5.50). The results of the Spearman test showed a positive correlation between GDM patients’ perception of future diabetes risk and postpartum medication adherence (r= 0.778, P<0.001). Additionally, self-efficacy was also positively correlated with medication adherence (r= 0.631, P<0.001). The results of the linear stratified regression model indicated that self-efficacy moderates the relationship between risk perception and medication adherence.

**Conclusion:**

The results of the MMAS-8 scale survey indicate poor medication adherence among postpartum GDM patients, with a significantly lower rate of good medication adherence compared to general T2DM patients. The stratified regression analysis demonstrates that risk perception and self-efficacy jointly influence postpartum medication adherence, and the risk perception attitude model can predict medication adherence among GDM patients postpartum.

## 1 Background

**Figure 1:**
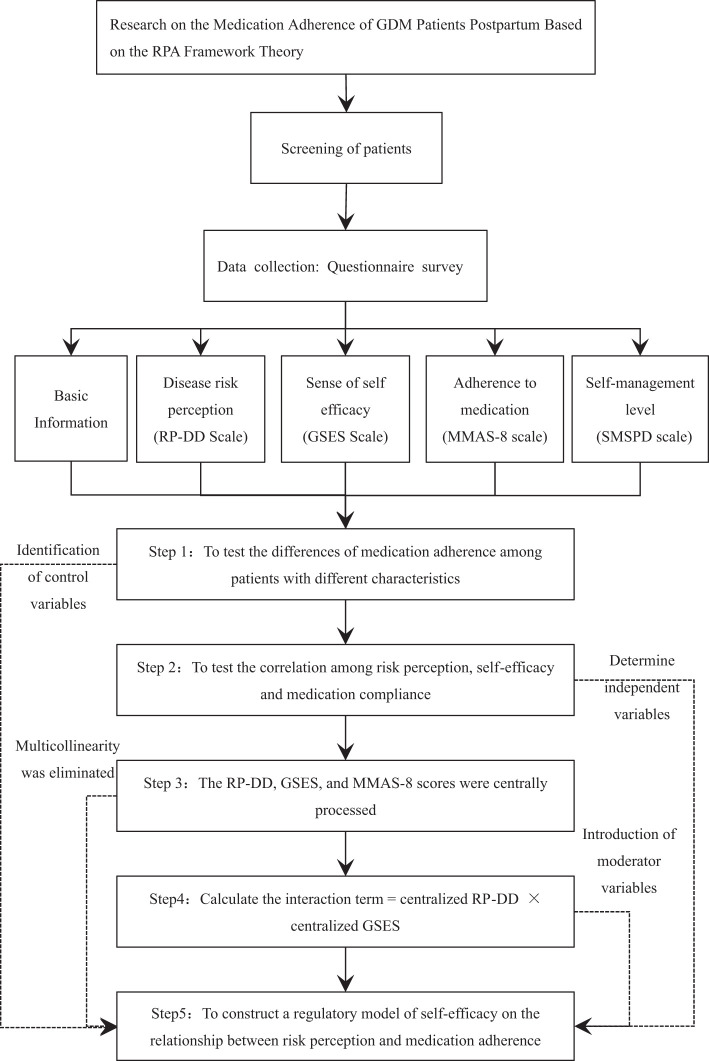
Technical roadmap for postpartum medication adherence research of GDM patients based on RPA framework theory.

In recent years, China’s family planning policy has undergone significant transformations. Implementing the “three-child” policy in 2021 has led to a yearly increase in the personal of pregnant women aged 35 and above (1–3). Consequently, the prevalence of pregnancy complications, such as Gestational Diabetes Mellitus (GDM), which is closely related to age, has risen significantly (4, 5). According to data from the International Diabetes Federation (IDF), the incidence rate of GDM in China was 8.6% in 2021 (6, 7). However, due to regional disparities in lifestyle and nutritional status, reports from different provinces reveal that the incidence rate of GDM ranges from approximately 5.33% to 19.7% (7–10). In addition, patients with gestational diabetes mellitus (GDM) also face long-term health risks. Some patients return to normal blood glucose levels after delivery, while others continue to have persistent abnormalities in glucose metabolism. Several investigations have indicated that a significant number of GDM patients exhibit varying degrees of abnormal glucose metabolism in the early postpartum period. Wu et al. (11)reviewed the results of glucose screening in 189 GDM patients from Jilin Province at 6 to 12 weeks postpartum. The findings showed that among patients who adhered to follow-up visits, 48 cases (29.2%) were diagnosed with prediabetes, and 13 cases (10.8%) had already progressed to Type 2 Diabetes Mellitus (T2DM). In another study conducted by Lin et al. (12) on 1362 GDM patients from Hubei Province, the results of glucose screening at 6 to 12 weeks postpartum revealed 271 cases (19.9%) of impaired glucose tolerance (IGT) and impaired fasting glucose (IFG), as well as 32 cases (2.3%) of overt diabetes. Similar findings have been reported in international studies, with investigations on the postpartum glucose status of GDM patients showing rates of IGT or IFG ranging from 13.0% to 32.1% and rates of T2DM ranging from 2.9% to 6.4% (13–16). Moreover, if timely intervention is not provided to these patients with abnormal glucose metabolism in the early postpartum period, their metabolic status may progressively worsen. A meta-analysis involving 28 studies and a total of 170,139 cases found that the risk of developing T2DM after GDM increases linearly with longer follow-up times, with an estimated annual increment of 9.6‰ (17). Given that GDM is a chronic condition necessitating long-term self-management and pharmacological intervention, optimal medication adherence is pivotal for achieving desired glycemic control. The World Health Organization (WHO) has emphasized that improving patient adherence to prescribed medications yields greater health benefits than the development of new treatment methods (18). Therefore, this study aims to assess postpartum adherence to hypoglycemic medications among GDM patients through a questionnaire-based approach and to analyze the associated influencing factors. The findings will assist clinical pharmacists in providing more targeted medication education to patients, thereby optimizing their medication behavior and glycemic management. The technical route is shown in **Figure 1**.

## 2 Methods

### 2.1 The risk perception attitude framework theory

**Figure 2:**
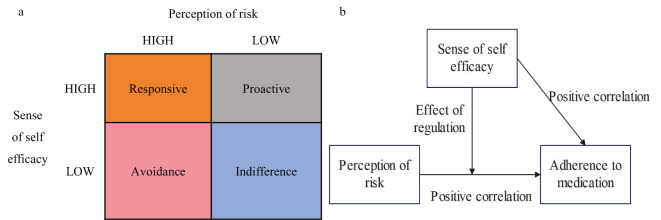
Framework of risk perception attitudes. **(a)** Grouping by Risk Attitude. **(b)** The Hypothesis of the Perceptual Framework of Risk Attitude.

The Risk Perception Attitude (RPA) framework theory was established by American scholar Rimal and colleagues in 2003, and it is used to predict public attitudes and behaviors when facing disease threats (19, 20). The fundamental hypothesis of this theory is that the perception of future disease risk and the sense of self-efficacy are the two main factors influencing whether the public will take preventive measures. Among these, risk perception serves as the motivational factor for preventive behavior, while efficacy beliefs act as the facilitating factor for such behavior. Therefore, based on the levels of these two influencing factors, the population can be divided into four risk attitude groups: Responsive, Avoidance, Proactive, and Indifference, as shown in **Figure 2A**. These different groups exhibit varying degrees of self-protective motivation, intentions to acquire knowledge and behavioral intentions. Individuals falling under the “Proactive” and “Independent” classifications in the RPA framework are less inclined to engage in active behavior modification, given their low perceived risk levels. The “Responsive” cohort, defined by high levels of both risk perception and self-efficacy, shows an increased inclination towards active health behavior changes. Meanwhile, the “Avoidance” group, despite high risk awareness, struggles with low confidence in their ability to implement changes. Compared to the “Responsive” group, they are less likely to take action to change their behavior.

Several studies have revealed that personalized intervention strategies based on the risk perception attitude framework can effectively enhance the self-efficacy of the target group, particularly exerting significant positive influences in diabetes prevention and health behavior management. For example, Rains et al (21).emphasized that personalized information dissemination is conducive to increasing the screening rate. Simonds et al (22). indicated that risk perception and self-efficacy are crucial factors influencing diabetes prevention. Zou Xiaohui (23) emphasized that in the prediabetic population, self-efficacy plays a regulatory role in health behaviors. Nevertheless, the study by Jones et al. (24) discovered that even if patients possess a high level of risk perception and health knowledge, these factors do not effectively enhance self-efficacy, especially in preventing cardiometabolic diseases after gestational diabetes. Overall, although the RPA framework holds certain guiding significance in improving patients’ health behaviors, in different populations and intervention contexts, a single risk perception and self-efficacy do not always fully account for the mechanism of behavioral change. These disparities suggest that the application effect of the RPA framework in diverse situations still requires further exploration and validation. By categorizing patients according to their risk attitudes, targeted interventions and educational materials can be provided to enhance glycemic control. However, there has been no specific research focusing on postpartum glycemic management in GDM patients. It is essential to fully understand how their perception of future diabetes risk and their sense of self-efficacy influence medication adherence, in order to optimize healthcare services.

To address this objective, the present investigation advances the following set of hypotheses (**Figure 2B**):

H1: There are significant differences in postpartum medication adherence among GDM patients with different individual characteristics
H2: Perceived risk is positively correlated with medication adherence;
H3: Self-efficacy is positively correlated with medication adherence;
H4: Perceived risk and self-efficacy jointly influence medication adherence;
H5: Self-efficacy moderates the relationship between perceived risk and medication adherence.

### 2.2 Study subjects

The study included a total of 80 participants. Participants were recruited from pregnant women with GDM registered in the obstetrics department of a tertiary hospital in China between January 1, 2020 and December 31, 2022.

The study enrolled patients who met the following criteria: (1) Patients who delivered in the obstetrics department of our hospital between 2020 and 2022 and were diagnosed with GDM during pregnancy; (2) Patients diagnosed with abnormal glucose metabolism during postpartum blood glucose screening conducted 4 to 12 weeks after delivery; (3) Patients who continued to use anti-hypoglycemic drugs for more than 3 months postpartum.

Exclusion criteria included: (1) Patients with severe comorbidities involving the heart, brain, lungs, kidneys, liver, or immune system; (2) Patients who failed to undergo follow-up blood glucose tests after being diagnosed with abnormal glucose metabolism postpartum; (3) Patients experiencing impaired consciousness, inability to communicate normally, or refusal to complete the questionnaire; (4) Patients with significantly incomplete records.

### 2.3 Morisky medication adherence scale survey

Approaches to evaluating medication adherence are commonly segregated into direct and indirect categories. The direct methods comprise observational techniques, physiological marker assessment, and blood drug level quantification. Indirect methods encompass scale evaluation, pill count, and tracking clinic visits, among others. Clinical researchers and medical practitioners frequently opt for the scale evaluation method, given its inherent simplicity, ease of administration, low resource requirements, and high operational feasibility. This study employed the 8-item Morisky Medication Adherence Scale (MMAS-8), developed by Professor Morisky in 2008 (25). The original MMAS-8 has a Cronbach’s α coefficient of 0.83. The Chinese version of the MMAS-8, translated by Wang Jie and others (26), has a reliability coefficient of 0.65 and a validity coefficient of 0.80, demonstrating good predictive validity. It has been extensively used by Chinese researchers to assess medication adherence in patients undergoing long-term diabetes treatment (27, 28).

Please note that, unlike the original MMAS-8 scale where a score of ≥8 indicates good adherence, we have made adjustments to the classification criteria for the total score of MMAS-8 in consideration of the characteristics of the participants and the nature of antidiabetic medication treatment. During the assessment process, it was observed that participants in this study, especially postpartum women with a history of gestational diabetes and recent abnormal glucose metabolism, exhibited extremely poor medication adherence due to various factors such as infant care, postpartum depression, inadequate medication education, and neglect of their own health. As a result, there was a severe differentiation in the scale scores and an uneven distribution. In this study, researchers first provided the respondents with a detailed explanation of the study’s purpose and the content of the scale. The respondents were then asked to truthfully answer the 8 items based on their recent medication behaviors (see **Table 1**). The scoring method is as follows: for items 1/2/3/4/6/7 a response of “Yes” is scored as 0, while “No” is scored as 1; item 5 is reverse scored;Item 8 is scored using a Likert 5-point scale, with scores assigned as 1/0.75/0.50/0.25/0, from highest to lowest. The total score for the 8 items is then calculated. A score of ≥6 indicates good adherence, 4 to 6 indicates moderate adherence, and <4 indicates poor adherence (30).

**Table 1:** 8-item morisky medication adherence scale^1^.

Items	The problem	Options
1	Did you sometimes forget to take diabetes medications?	Yes (0) No (1)
2	Over the past two weeks, were there any days when you did not take diabetes medicine?	Yes (0) No (1)
3	Have you ever cut back or stopped taking your diabetes medication without telling your doctor because you felt worse when you took it?	Yes (0) No (1)
4	When you travel or leave home,do you sometimes forget to bring along your diabetes medications?	Yes (0) No (1)
5	Did you take your diabetes medicine yesterday?	Yes (1) No (0)
6	When you feel like that your diabetes is under control,do you sometimes stop taking your medicine?	Yes (0) No (1)
7	Do you feel hassled about sticking to your diabetes treatment plan?	Yes (0) No (1) Never (1) Occasionally (0.75)
8	Do you have difficulty remembering to take your medication?	Sometimes (0.5) often (0.25) All the time (0)

^1^©MMAS 2006 www.adherence.cc.

### 2.4 Analysis of the moderating effect of self-efficacy on the relationship between risk perception and medication adherence

#### 2.4.1 Variable collection

In order to improve the feasibility of the questionnaire and the quality of the data, the basic information of patients will be automatically extracted by the researchers from the HIS system, including age, childbirth history, mode of delivery, adverse pregnancy outcomes, severe comorbid diseases, family history of diabetes, blood glucose management methods during pregnancy, medication in formation, and laboratory examination data. The content that needs to be filled in by the patients themselves includes the receipt of prenatal and postnatal health education, the average monthly income per capita of the family, the payment method for medical expenses, the care of the baby by family members, po stpartum depression, the situation of postpartum overweight or obesity, and the duration of breastfeeding. The perception of disease risk, self-efficacy, and self- management level will be investigated using the RP-DD scale(Risk Perception f or Developing Diabetes) (29), the GSES scale(Generalized Self-Efficacy Scale, GSES) (30), and the SMSPD scale(Self-management Scale for Prediabetes) (31), respectively.

Disease Risk Perception Scale: This study utilized the Risk Perception for Developing Diabetes (RP-DD) scale, developed by American scholar Walker in 2003 (29, 32), to measure the perceived risk of developing diabetes in individuals with high-risk factors or pre-diabetes. The specific content is detailed in Appendix 1.

Self-Efficacy Scale: This study utilized the Generalized Self-Efficacy Scale (GSES), developed by German scholar Schwarzer based on self-efficacy theory (30). The GSES consists of 10 items (see Appendix 2) and is scored using a 4-point Likert scale, with a total score range of 10 to 40 points. Higher scores indicate stronger self-efficacy in individuals.

Self-Management Scale: Given that the study population predominantly includes patients with Impaired Glucose Tolerance (IGT) or Impaired Fasting Glucose (IFG), the Self-management Scale for Prediabetes (SMSPD) was used. This scale, developed by Chinese scholar Ge Guo et al. (31, 33) based on the framework of social cognitive theory, has a Cronbach’s α coefficient of 0.892 (see Appendix 3).

In order to optimize the questionnaire design, this study preliminarily determined the questionnaire content through the combination of previous literature review and expert consultation, and adopted convenience sampling to randomly select 50 patients with gestational diabetes mellitus and postpartum abnormal glucose metabolism meeting the exclusion criteria for pre-experiment. The filling rate, filling time and the reasons for not completing the filling were analyzed. The results showed that only 58.0% of the patients completed all questionnaires, and the average filling time was 16.3 ± 4.23 minutes. The main reasons for incomplete filling include: heavy filling burden due to a large number of questions, low correlation between the dimensions of “environmental risk” and “risk of other diseases” in RP-DD scale and daily life, and some items of the dimensions of “health concept” and “self-efficacy” in SMSPD scale are highly similar to other scales, with redundancy. Based on the above results, in order to improve the questionnaire completion rate and reduce the burden on respondents, the RP-DD and SMSPD scales were deleted and modified accordingly after expert discussion, and the final questionnaire was formed for formal research. There are 5 doctors, nurses and clinical pharmacists who have been working in endocrinology for more than 5 years and have rich experience in clinical drug treatment, forming an expert consulting group with reasonable discipline structure.

#### 2.4.2 Statistical testing

All the data obtained from HIS system and questionnaire survey were recorded into Microsoft Excel 2023 for summary and processing, and then imported into IBM SPSS 26.0 for description and analysis. For continuous variables, Shapiro-Wilk normality test was performed first. The mean ± standard deviation was used to represent the normal distribution, and the independent sample t test was used for the difference between groups. The median (lower quartile, upper quartile) that did not meet the normal distribution was represented by non-parametric tests, including Mann-Whitney U rank sum test for differences between 2 independent samples and Kruskal-Wallis test for differences between more than 2 independent samples. In all statistical tests, bilateral P < 0.05 was considered statistically significant.

First, the difference in the total score of MMAS-8 of 20 basic characteristics in the study population corresponding to different levels was compared to test the difference in medication compliance of patients with different characteristics (addressing hypotheses H1).

Next, the investigation explored the associations among disease risk perception, self-efficacy, and medication adherence (addressing research hypotheses H2 and H3). Spearman’s correlation test was conducted on the total scores of the RP-DD, GSES, SMSPD, and MMAS-8, which are all continuous variables. A correlation matrix was constructed, with a correlation coefficient of *r* > 0 and a *P*-value < 0.05 set as the criteria for determining a positive correlation between two variables.

The final phase of the investigation examined self-efficacy’s potential moderating role in the association between risk perception and medication adherence (addressing research hypotheses H4 and H5). In the moderation analysis model, the moderating variable is the third variable that can influence the direction and strength of the relationship between the independent and dependent variables. In this study, the control variables are the patient characteristics that significantly impacted medication adherence in the first step of the analysis. The independent variables are the total RP-DD score, the total GSES score, and their interaction term, while the dependent variable is the total MMAS-8 score. The specific testing steps are as follows: First, the total scores of the RP-DD, GSES, and MMAS-8 were mean-centered (i.e., centered value = original value − mean value) to eliminate severe multicollinearity between the independent variables. Then, an interaction term was constructed (interaction term = centered RP-DD total score × centered GSES total score). Finally, a hierarchical linear regression model was established. The control variables were entered into the first layer of the regression equation. The centered RP-DD total score and the centered GSES total score were entered into the second layer. The interaction term was entered into the third layer. The changes in the model’s R² were observed as different variables were added, as well as the significance of each variable. If the change in R² is significant with the addition of the interaction term, and the interaction term itself is significant, this indicates that the moderating effect is established.

## 3 Results

### 3.1 Patient characteristics

A total of 2765 patients with GDM who were recorded and delivered in our hospital during 2020~2022 were retrospectively collected in HIS system. Of the 1153 (41.7%) patients who returned to the hospital at 4 to 12 weeks postpartum for blood glucose screening, 619 (22.4%) underwent a complete 75g OGTT and 534 (19.3%) had only FBG and/or HbA1c tested. Among the patients who underwent blood glucose screening 4 to 12 weeks postpartum, 425 (36.9%) had abnormal glucose metabolism. After excluding patients with severe diseases (9 patients), no blood glucose review (72 patients), refusal to fill in the questionnaire (47 patients), and serious data missing (23 patients), 80 of the remaining 274 patients received hypoglycemic drug treatment lasting more than 3 months after delivery and were included in the study.

A total of 80 postpartum GDM patients who received pharmacological interventions were surveyed to assess their medication adherence. The median score on the MMAS-8 scale was 3.75 (3.50, 5.50). Among these patients, 11 (13.8%) exhibited good adherence, 28 (35.0%) showed moderate adherence, and 41 (51.3%) had poor adherence. Significant differences in medication adherence were observed among patients with different educational levels, postpartum health education status, and treatment regimens, with these differences being statistically significant (*P* < 0.05), thereby confirming research hypothesis H1. Detailed data are presented in **Table 2**.

**Table 2:** Basic characteristics and medication adherence of GDM patients.

Characteristics of patients		MMAS-8 medication adherence score	Z/H	P
Disease status at the time of medication initiation	Prediabetes T2DM	3.88 (3.50, 4.69) 3.75 (3.25, 5.00)	-0.091	0.927
Interval between delivery and initiation of medication	<12 months 12~18 months ≥18 months	4.50 (3.75, 5.25) 3.75 (3.25, 4.50) 3.75 (3.25, 5.00)	3.241	0.198
hypertension	No Yes	3.88 (3.25, 5.00) 3.75 (3.75, 4.94)	-0.829	0.407
Dyslipidemia	No Yes	3.75 (3.25, 5.00) 4.38 (3.50, 5.00)	-0.672	0.502
Family history of diabetes	No Yes	3.75 (3.25, 4.88) 3.75 (3.50, 5.00)	-0.351	0.725
Whether it was an advanced maternal age	No Yes	4.25 (3.50, 5.00) 3.75 (3.06, 4.81)	-1.29	0.197
History of delivery	First birth multiparity	3.75 (3.50, 4.56) 4.13 (3.06, 5.13)	-0.512	0.609
Mode of delivery	Natural birth Cesarean section	4.00 (3.25, 4.88) 3.75 (3.50, 5.00)	-0.132	0.895
Whether there were adverse pregnancy outcomes	No Yes	4.00 (3.00, 5.25) 3.75 (3.50, 4.50)	-0.407	0.684
The presence or absence of postpartum obesity	normal Overweight Obesity	4.13 (3.50, 5.50) 4.00 (3.75, 4.88) 3.75 (2.25, 4.50)	2.601	0.272
Education background	High school and below Junior college Undergraduate Graduate student or above	2.50 (3.50, 3.75) 3.50 (3.75, 5.00) 3.25 (3.75, 4.75) 3.75 (4.50, 5.50)	8.384	0.039
Payment methods for medical expenses	Urban workers or urban and rural residents Business insurance Out of pocket	3.75 (3.25, 5.13) 4.50 (4.25, 6.00) 3.75 (3.38, 4.00)	4.994	0.082
Per capita monthly household income	<5000 5000~10000 ≥10000	3.50 (2.75, 3.75) 3.75 (3.63, 5.25) 4.50 (3.50, 5.00)	4.062	0.131
Health education during pregnancy	No Yes	3.75 (2.50, 5.75) 4.00 (3.50, 5.00)	-0.36	0.719
Postpartum health education	No Yes	2.38 (3.50, 3.88) 3.75 (4.50, 5.50)	-3.562	<0.001
Blood glucose management during pregnancy	Nonpharmacological interventions Pharmacological interventions	3.88 (3.50, 4.50) 3.75 (3.25, 6.13)	-0.754	0.451
Postpartum depression	No Yes	3.88 (3.25, 5.00) 3.75 (3.50, 4.63)	-0.19	0.85
Whether there are family members Help take care of the baby	No Yes	4.25 (3.38, 5.50) 3.75 (3.50, 5.00)	-0.254	0.799
Treatment Options	Monotherapy Dual Therapy	3.25 (3.75, 4.50) 3.75 (4.75, 6.50)	-2.191	0.028
Route of Administration	Oral administration Subcutaneous injection	3.75 (3.50, 5.00) 4.00 (2.38, 5.38)	-0.018	0.986

### 3.2 Correlation between risk perception, self-efficacy, and medication adherence

The correlation matrix for the total scores of RP-DD, GSES, SMSPD, and MMAS-8 is shown in **Table 3**. Spearman’s test results indicate that the perception of future diabetes risk among GDM patients is positively correlated with postpartum medication adherence (*r* = 0.778, *P* < 0.001), confirming research hypothesis H2. Additionally, self-efficacy was also positively correlated with medication adherence (*r* = 0.631, *P* < 0.001), confirming research hypothesis H3. Furthermore, a positive correlation was found between risk perception and self-efficacy (*r* = 0.241, *P* = 0.031), while no significant correlation was observed between self-management levels and the other three variables (*P* > 0.05).

**Table 3:** Correlation matrix.

Spearman correlation test		RP-DD	SMSPD	GSES	MMAS-8
RP-DD	r *P*	- -	0.162 0.152	0.241 0.031	0.778 <0.001
SMSPD	r *P*	0.162 0.152	- -	0.205 0.067	0.194 0.084
GSES	r *P*	0.241 0.031	0.205 0.067	- -	0.631 <0.001
MMAS-8	r *P*	0.778 <0.001	0.194 0.084	0.631 <0.001	- -

### 3.3 The moderating effect of self-efficacy on the relationship between risk perception and medication adherence

A linear hierarchical regression model was selected to examine the moderating effect.

In the initial Model 1, only three control variables were included: patient education level, whether postpartum health education was received, and the treatment regimen. In Model 2, with the addition of independent variables, the significance of the three control variables diminished, while the centered total scores of RP-DD and GSES became significant. This indicates that both risk perception and self-efficacy jointly influence patients’ medication adherence, confirming research hypothesis H4. Model 3 further introduced an interaction term based on Model 2. The change in R² was significant, and findings showed a meaningful interaction, suggesting that self-efficacy moderates how risk perception affects an individual’s adherence to prescribed medications, thereby confirming research hypothesis H5. The model parameters are detailed in **Table 4**.

**Table 4:** Multilevel linear regression.

Variables of interest	Model 1			Model 2			Model 3		
	β	*P*	VIF	β	*P*	VIF	β	*P*	VIF
Education background	0.331	0.091	1.050	0.003	0.968	1.194	-0.034	0.670	1.215
Treatment Options	0.802	0.068	1.019	0.349	0.055	1.043	0.244	0.154	1.076
Postpartum health education	1.090	0.006	1.069	0.043	0.802	1.250	0.034	0.828	1.250
Centralized RP-DD	–	–	–	0.392	<0.001	1.104	0.407	<0.001	1.140
Centralized GSES	–	–	–	0.214	<0.001	1.447	0.232	<0.001	1.508
Term of interaction	–	–	–	–	–	–	0.025	0.001	1.116
Term of constant	-1.828	0.002	–	-0.108	0.688	–	-0.044	0.861	–
ΔR^2^		0.201			0.668			0.020	
ΔF		6.376			189.364			12.903	
*P*		0.001			<0.001			0.001	

**Figure 3:**
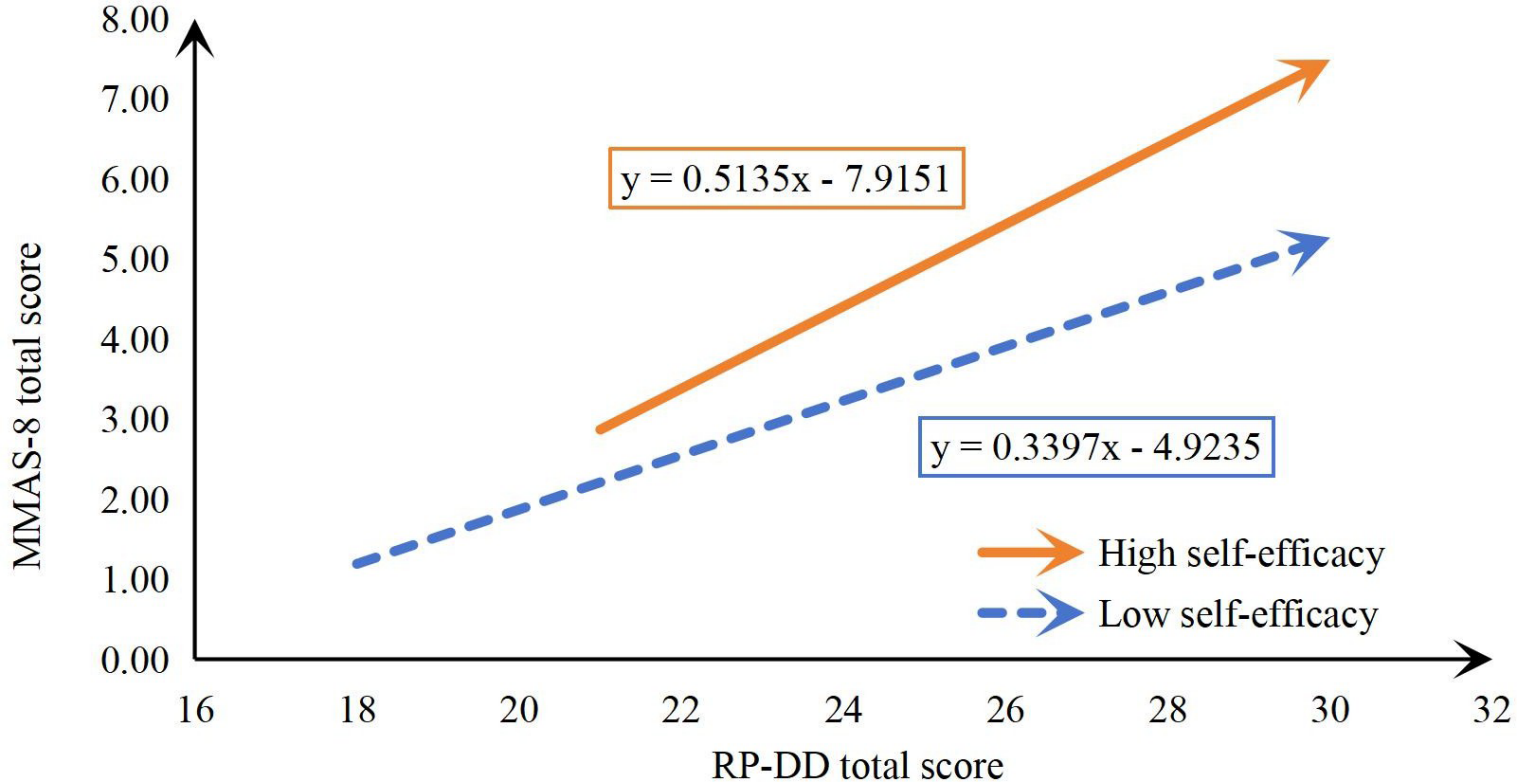
The moderating effect of self-efficacy on the relationship between risk perception and medication adherence.

To visually represent this moderating effect, patients were categorized into two groups – those with high and low self-efficacy – determined by their GSES scale scores, with a threshold of 24 points. A moderation effect plot was created, with RP-DD total score on the x-axis and MMAS-8 total score on the y-axis (**Figure 3**). As shown in the figure, irrespective of self-efficacy grouping, findings indicate a propensity for risk perception to correlate positively with adherence to medication protocols; Nevertheless, the high self-efficacy group demonstrated a markedly steeper gradient in the trend line compared to their low self-efficacy counterparts. This indicates that higher self-efficacy enhances the positive predictive effect of risk perception on medication adherence. Conversely, when self-efficacy is lower, this positive effect is diminished.

## 4 Discussion

In the long-term pharmacological management of diabetes patients, maintaining a high level of medication adherence is crucial to achieving the desired glycemic control outcomes. This section of the study builds upon the earlier exploration of postpartum medication behaviors in patients with gestational diabetes mellitus (GDM) and further analyzes medication adherence and its related factors.

### 4.1 Discussion on medication adherence survey results

Quantification of adherence levels among study participants was accomplished through the application of the 8-item Morisky Medication Adherence Scale (MMAS-8). The maximum possible score on the MMAS-8 scale is 8 points; however, the median score among patients in this study was only 3.75 (3.50, 5.50). Defining good adherence as a score of ≥6 points, only 13.8% of the patients in this study met this criterion, which is significantly lower than the levels reported in other studies. For instance, in the study conducted by Wang Jie et al. (26), 73.1% of T2DM patients met the criterion for good adherence, while in the studies by Yu Yebo et al. (27) and Wu Ping et al. (28), the rates were 58.7% and 53.0%, respectively. These data suggest that, compared to general T2DM patients, GDM patients exhibit a significant deficiency in postpartum medication adherence. One possible explanation for this discrepancy might be that individuals might be experiencing the initial phases of the illness and have not yet encountered the typical symptoms and complications as with diabetes, leading to a diminished emphasis on the regularity and consistency of glycemic control treatment.

### 4.2 Discussion on the moderating role of self-efficacy on diabetes risk perception and medication adherence

Based on the RPA framework theory, this study analyzed the impact of diabetes risk perception and self-efficacy on the postpartum medication adherence of patients with gestational diabetes mellitus (GDM). The results of the Spearman test showed that both the risk perception of future diabetes (r = 0.778, P < 0.001) and self-efficacy (r = 0.631, P < 0.001) of GDM patients were positively correlated with medication adherence. The results of further hierarchical linear regression indicated that self-efficacy played a moderating role in the impact of risk perception on medication adherence. Jones et al. (24) found that although some groups of people (such as American Indian women) have a high level of disease awareness and risk perception, if they lack self-efficacy, they may still find it difficult to take action, which is similar to the performance of the low self-efficacy group in this study. The study by Anahita Babak et al. (34) on the impact of self-efficacy training on patients with type 2 diabetes mellitus showed that self-efficacy training effectively improved the metabolic control of patients with type 2 diabetes mellitus and, to a certain extent, had good medication adherence.

In addition, previous research results have shown that the factors affecting the medication adherence of diabetes patients include socioeconomic status, the complexity of the treatment regimen, drug side effects, patients’ awareness of the disease and treatment, and the accessibility of medical resources. This partially coincides with the results observed in our univariate analysis, that is, patients with different educational backgrounds, those who received or did not receive postpartum health education, and those using different treatment regimens showed significant differences in medication adherence (P < 0.05). However, in the multivariate analysis, the impact of these three patient characteristics on medication adherence was not statistically significant. It may be because the effects of educational background, postpartum health education, and treatment regimen on medication adherence were masked by the effects of risk perception and self-efficacy. It is necessary to further examine the differences in risk perception and self-efficacy among patients with different characteristics and gain an in-depth understanding of the significance of these differences for improving patients’ medication adherence and optimizing blood glucose management.

This study still has certain limitations: (1) The sample size included in the study is relatively small (80 cases); (2) The single-center research design implies that the geographical distribution of the study population is relatively concentrated, and the conclusions should be interpreted with caution when extended to populations in different regions or ethnic groups; (3) Although a pre-experiment was conducted to optimize the questionnaire design, it is still impossible to completely eliminate the bias caused by participants’ poor understanding of the questions.

## 5 Conclusion

This study focused on the medication adherence of patients with GDM and those experiencing postpartum glucose metabolism abnormalities. Additionally, it examined how risk perception and self-efficacy influenced their adherence to medication regimens among these patients. The results of the MMAS-8 scale survey indicated that postpartum medication adherence among GDM patients is suboptimal, with the rate of optimal adherence being significantly lower than that of general T2DM patients. Stratified regression analysis showed that risk perception and self-efficacy jointly influence postpartum medication adherence, and the Risk Perception Attitude (RPA) model can predict medication adherence in GDM patients postpartum.

Therefore, clinical pharmacists should place greater emphasis on patients with gestational diabetes. Through systematic health education, ongoing support, and encouragement, the goal should be to enhance pregnant women’s awareness of their disease risk and to improve their self-efficacy. Such endeavors are anticipated to enhance medication adherence, ultimately leading to better glycemic control.

## Data Availability

The original contributions presented in the study are included in the article/supplementary material. Further inquiries can be directed to the corresponding authors.
